# The Influence of Different Aggregates on the Physico-Mechanical Performance of Alkali-Activated Geopolymer Composites Produced Using Romanian Fly Ash

**DOI:** 10.3390/ma17020485

**Published:** 2024-01-19

**Authors:** Adrian-Victor Lăzărescu, Andreea Hegyi, Alexandra Csapai, Florin Popa

**Affiliations:** 1 NIRD URBAN-INCERC Cluj-Napoca Branch, 117 Calea Florești, 400524 Cluj-Napoca, Romania; adrian.lazarescu@incerc-cluj.ro; 2 Faculty of Materials and Environmental Engineering, Technical University of Cluj-Napoca, 103-105 Muncii Boulevard, 400641 Cluj-Napoca, Romania; florin.popa@stm.utcluj.ro

**Keywords:** geopolymer composites, fly ash, micronized quartz, glass aggregates

## Abstract

In light of the urgent need to develop environmentally friendly materials that, at some point, will allow the reduction of concrete and, consequently, cement consumption—while at the same time allowing the reuse of waste and industrial by-products—alkali-activated fly ash (AAFA) geopolymer composite emerges as a material of great interest. The aim of this study was to investigate the physico-mechanical performance of composites based on AAFA binders and the effect of different types of aggregates on these properties. The experimental results indicate variations in flexural and compressive strength, which are influenced both by the nature and particle size distribution of aggregates and the binder-to-aggregate ratio. The analysis of the samples highlighted changes in porosity, both in distribution and pore size, depending on the nature of the aggregates. This supports the evolution of physico-mechanical performance indicators.

## 1. Introduction

Cement production contributes at least 5–8% of global carbon dioxide emissions [1]. Such a massive output has a significant impact on the environment. A sustainable alternative to cement-intensive concrete is geopolymer binders [2,3,4]. These are currently under development, and research is focused on meeting the imperative to reduce global CO_2_ emissions. With excellent mechanical properties and durability in challenging environments, these materials offer an opportunity for both environmental and engineering considerations, providing an alternative to conventional technology [5,6]. Geopolymer concrete is considered a third-generation binder after lime and cement. Some studies using the European life cycle database, Ecoinvent, suggest that using it may lead to a potential reduction in greenhouse gas emissions ranging from 25 to 45% [7], or even up to 70% [8].

Geopolymer binders are essentially formed through chemical reactions. The specific category is created by the alkaline activation of materials abundant in SiO_2_ and Al_2_O_3_ [9]. The ash from thermal power plants contains significant proportions of aluminium and amorphous silica, making it a suitable source for geopolymer production [10]. The steps of the chemical process used to obtain geopolymers by alkaline activation of the thermal power plant ash, as outlined by Buchwald et al. [11], can be described by the chemical reaction. The overall chemical reaction of thermal power plant ash is expressed by Equation (1). As a result of this repolymerization mechanism, a distinct spatial, three-dimensional arrangement can be observed at a microstructural level in geopolymers; this is in contrast to the non-spatial arrangement of Si and Ca oxides typically found in cementitious composites [12].

 SiO_2_ · αAl_2_O_3_ · βCaO · γNa_2_O · δFe_2_O_3_ · εTiO_2_ + (β + γ + 3δ)H_2_O + (2 + 2α + ε)OH → SiO_3_²⁻ + 2αAlO²⁻ + βCa²⁺ + 2γNa⁺ + 2δFe³⁺ + εHTiO³^−^ + (1 + α)H_2_O + 2(β + γ + 3δ) (1)

Therefore, as reported in the literature, the microstructural characteristics of geopolymer composites are closely related to the specific raw materials and the production technology [4,9,13,14,15,16,17,18]. These significantly influence the physico-mechanical performance and durability of the cured and matured composite [19,20,21,22,23,24,25,26,27,28,29,30,31,32,33,34,35,36,37,38,39,40,41,42,43,44,45,46,47,48,49,50,51,52,53,54,55,56,57,58,59,60,61,62,63,64,65,66,67,68,69,70,71,72].

Several studies have documented a wide variation in the oxide composition of fly ash; the main oxides are Al_2_O_3_, SiO_2_, Fe_2_O_3_ and CaO. The prevailing consensus is that fly ash consists predominantly of spherical, amorphous particles, but it also contains crystalline structures that dissolve more gradually and often only partially during the geopolymerization process. In addition, it is acknowledged in the literature that these oxides are present in fly ash in different mineralogical phases such as anhydrite, quartz, portlandite, hematite, calcite or, to a lesser extent, mullite. The reactivity of fly ash tends to be higher when the content of crystalline phases is lower [10].

When produced from various raw materials, geopolymers typically consist of a blend of crystalline aluminosilicate particles and semicrystalline and amorphous aluminosilicate gels. A detailed characterization of their structural constitution is challenging because of their complex composition and the difficulty in separating the crystalline aluminosilicate particles from the semi-crystalline and amorphous gel phases; however, this is vital to understand their mechanical strength. Therefore, a comprehensive understanding of both the structural composition and the gel phases in geopolymers is essential [73]. Scanning electron microscopy (SEM) coupled with energy-dispersive spectroscopy (EDS) stands out as a primary method employed for the analysis of microstructures. These methods have been previously employed for the characterization of clay, zeolites, fly ash, cement and concrete [73,74,75,76,77,78,79,80,81].

Consequently, the literature points to a significant variation in the mechanical strength of both the geopolymer binder and geopolymer composites (which consists of the binder matrix containing the aggregate skeleton). The variability is influenced by factors such as the characteristics and percentage content of Si and Al in the fly ash, the Si/Al ratio, the type of alkaline activator used, the molarity of the NaOH or KOH solution used, the mass ratio between the Na_2_SiO_3_ and NaOH (or KOH) solutions in the preparation of the alkaline activator, the duration and temperature of the heat treatment or the age at testing. Compressive strengths in the range of 10 MPa to 95 MPa have been reported for both the geopolymer binder and the geopolymer composite, and aggregate granulation up to 4 mm has been observed [19,20,21,22,23,24,25,26,27,28,29,30,31,32,33,34,35,36,37,38,39,40,41,42,43,44,45,46,47,48,49,50,51,52,53,54,55,56,57,58,59,60,61,62,63,64,65,66,67,68,69,70,71,72]. Research has shown that heat treatment temperature is critical in formulating and producing geopolymer materials. The ideal temperature for heat treatment falls between 60 °C and 75 °C [61].

There are limited studies about the use of different sands as aggregates in the geopolymer mortar. Aggregates greatly influence the characteristics of mortar or concrete, both in fresh and hardened states. Their grading, shape and texture greatly affect properties of concrete in a fresh state (i.e., workability, finishability, bleeding or segregation). Moreover, when hardened characteristics are considered, density, mechanical strength, porosity or water absorption are also highly affected by aggregate features [82].

Several researchers have studied the influence of different types of aggregates in terms of mechanical properties of geopolymer materials [82,83,84,85,86]. Mechanical properties of geopolymer concrete with different fine aggregate content and grading (sand and granite slurry) were mixed together in different proportions (100:0, 80:20, 60:40 and 40:60) using fly ash and granulated slag as raw material, with a 50:50 aggregate:binder ratio [83]. The mechanical properties of the geopolymer materials (compressive strength, flexural strength) were studied after 7, 28 and 90 days of curing at ambient room temperature. The results show that the mechanical properties increased up to a fine aggregate proportion of 60:40; a decreasing trend has been observed at a proportion of 40:60.

Other studies have investigated the influence of aggregate mass percentages in the geopolymer binder to determine the impact of the ratio of binder:aggregate on the synthesized material properties [84]. The study shows that the incorporation of aggregates in the reaction mixtures changes the aspect of the materials due to interactions between binders and aggregates. Several other parameters that could influence adhesion between the aggregates and the binder are the porosity, the roughness of the aggregates and the chemical composition at the interface [85,86]. The optimal aggregate choice may vary depending on the specific application, desired properties and local material availability. Additionally, proper mix design and testing should be conducted to ensure the desired performance of geopolymer-based materials with selected aggregates.

The aim of this study is to analyse the effects of mixing different local source aggregates with an alkali-activated fly ash-based geopolymer binder in composite materials. These aggregates are sourced from either recycled waste (i.e., glass waste, spent garnet) or quartz aggregates; each is characterized by a different granulation that influences the basic physico-mechanical properties of the material.

## 2. Materials and Methods

### 2.1. Preparation of the Geopolymer Binder

**Figure 1:**
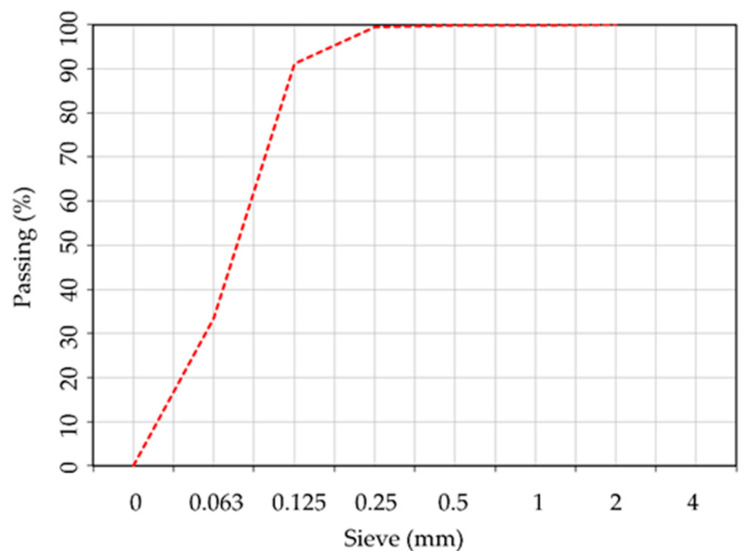
Fly ash particle size distribution.

**Figure 2:**
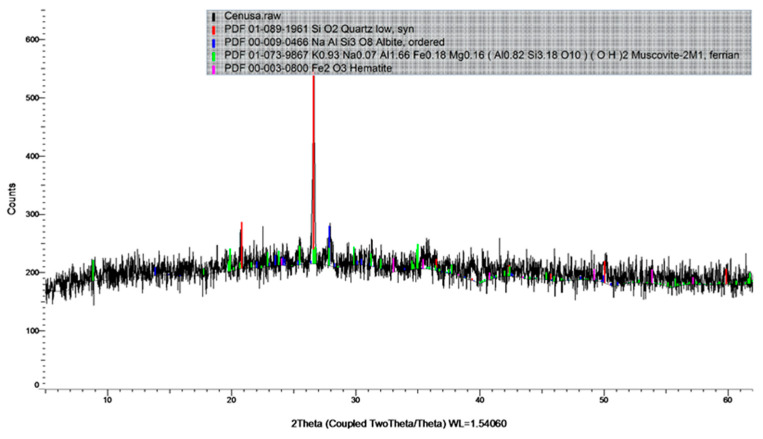
Screenshot of the XRD spectra for the fly ash powder.

The geopolymer binder was obtained by alkaline activation of fly ash (FA) obtained from Rovinari power plant in Romania. Fly ash was chosen because previous studies [87] have shown that its chemical composition and particle size distribution make it suitable for the production of geopolymer binders with increased mechanical performances. Characterization of the fly ash was carried out prior to its use in the preparation of the geopolymer binder. This included the determination of the chemical composition by XRF analysis and the assessment of the R_0.045_ fineness (Table 1 and Figure 1). The chemical composition and the particle size distribution of the fly ash were investigated using X-ray fluorescence (XRF) analysis, using a HELOS RODOS/L, R5 instrument (Sympatec GmbH, Clausthal-Zellerfeld, Germany) (Table 1). The mineralogical composition of the fly ash was investigated using an X-ray diffraction (XRD) analysis (Figure 2), Bruker D8 ADVANCE X-ray diffractometer (Bruker, Karlsruhe, Germany). Scans were collected in the range of 5–60° (2θ) with a step size of 0.02° and a scan speed of 10 s per step.

The diffuse reflection peak for quartz was predominantly at 26.59° (2θ), with additional smaller peaks at 20.82 and 50.14° 2θ. Albite was detected at 13.88, 23.53 and 27.90° (2θ). Muscovite M1 was identified at various angles: 19.91, 22.93, 25.50, 26.78, 27.90, 29.84 and 35.06° (2θ). Hematite was identified by X-ray diffraction at angles of 24.02, 33.09, 35.47, 40.82, 49.28, 53.96 and 57.29° (2θ). The peak with the highest intensity at 2θmax, 26.59° (2θ) was assigned to quartz.

Based on previous studies regarding the production of alkali-activated fly ash-based geopolymer binders [87], the alkaline activator solution (AA) was prepared by mixing a sodium hydroxide (NaOH) solution with a molar concentration of 8 M in a 1:1 constant mass ratio with a commercially purchased sodium silicate (Na_2_SiO_3_) solution at room temperature. After preparation and before use, the alkaline activator solution was stored in a sealed container under laboratory conditions (23 °C) for 24 h to mature.

The mixing of the components and the preparation of the geopolymer binder were carried out in a laboratory environment (23 °C and 65% RH) using an ELE laboratory mixer, with a stainless-steel beater (ELE International, Milton Keynes, UK), according to EN 196-1 [88]. The mass ratio between the amount of fly ash and the amount of alkaline activator used in the preparation was kept constant at 0.9.

The geopolymer binder sample, identified by code P1, was used as a control sample in comparison to all subsequent composite samples and was prepared using only fly ash and alkaline activator. The control sample, i.e., the binder sample, was prepared without aggregates, because the aim of the study was to comparatively analyse both the influence of different types of aggregates on the performance of the geopolymer material and the influence of introducing aggregates into the geopolymer binder, thus obtaining the transition to geopolymer mortar composites.

### 2.2. Preparation of the Geopolymer Composite Samples

**Figure 3:**
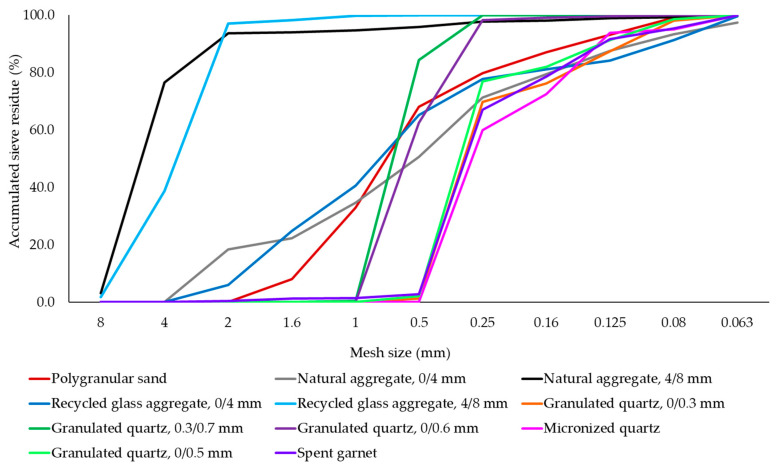
Graphical representation of the particle size distribution of the aggregates used in the production of alkali-activated fly ash-based geopolymer samples.

For the formulation of geopolymer composites, different types of aggregates with a maximum particle size of 8 mm have been incorporated into the geopolymer binder during the preparation process, with specific identification codes (P2–P12) for each mixture. These include polygranular CEN- NORMSAND EN 196-1 sand (P2), granular class 0/4 mm natural aggregates (P3), granular class 4/8 mm natural aggregate (P4), granular class 0/4 mm recycled glass aggregate (P5), granular class 4/8 mm recycled glass aggregate (P6), micronized quartz (P7), 0/0.3 mm granulated quartz (P8), 0/0.5 mm granulated quartz (P9), 0/0.6 mm granulated quartz (P10), 0.3/0.7 mm granulated quartz (P11) and spent garnet (P12). For each type of aggregate used in the production of the samples, bulk apparent density was analysed in accordance with EN 1097-3 [89] (Table 2); and particle size distribution (Figure 3) was analysed using a sieving method. This range of aggregates was chosen to facilitate an analysis of their influence on the physico-mechanical properties of the geopolymer composite, considering both the nature of the aggregate and the granularity characteristics.

To analyse both the influence of the type of aggregates used in the production of the geopolymer samples and the influence of mass ratio between geopolymer binder and aggregates on the physico-mechanical properties samples, three binder:aggregate ratios were used (1:1, 0.75:1 and 1.25:1). The components were mixed and the geopolymer composites were prepared under the same temperature, relative humidity and equipment conditions as those described in Section 2.1. A minimum of 3 sets of samples were produced to analyse the influence of the type, specific granulation and geopolymer binder/aggregate ratio on the physico-mechanical performances of the alkali-activated fly ash-based geopolymer mixtures.

**Figure 4:**
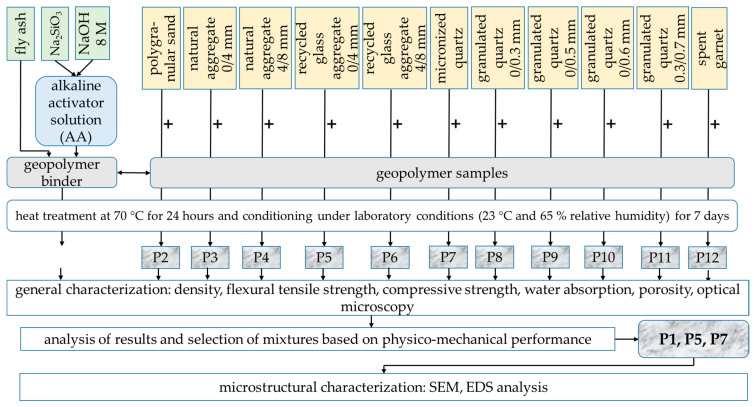
Graphical representation of the work flow for the preparation and analysis of the geopolymer recipes.

After being cast into 40 × 40 × 160 mm moulds, with the corresponding vibration, the samples were subjected to a (70 °C for 24 h) heat treatment using a thermostatic MEMMERT ULE 500 chamber (MEMMERT GmbH+Co.KG, Schwabach, Germany). After demoulding, the geopolymer samples were stored in laboratory conditions (T = 23 °C and RH = 65%). Testing to determine their mechanical properties was carried out after 7 days. The experimental research methodology is graphically outlined in Figure 4.

### 2.3. Physico-Mechanical Analysis of the Alkali-Activated Geopolymer Samples

Prior to the physico-mechanical performance evaluation tests, the density of the geopolymer samples was determined as the ratio between the mass of the samples, determined by weighing using a precision balance (KERN FKB 36K0.1, KERN & SOHN, Albstadt, Ebingen, Germany) and their volume, by direct measurement of the real dimensions using an electronic calliper with an accuracy of 0.01 mm.

To obtain the flexural and compressive strength results for all of the alkali-activated fly ash based geopolymer samples, tests were conducted according to the (EN 196-1:2006 [88]) standard method for evaluation of mechanical performances of OPC paste and standard type mortar.

Total water absorption of the alkali-activated fly ash-based geopolymer samples was determined by submerging the test samples in water, at a constant temperature of (20 ± 5) °C, until they reached constant mass. The minimum immersion period of the samples was 3 days. After reaching the saturated state constant mass, they were weighed. Subsequently, samples were placed in an oven and dried at a temperature of (105 ± 5) °C, to reach the constant mass. The water absorption of each sample was expressed as mass percentage loss.

Prior to the water absorption test, porosity was evaluated using a calculation for all alkali-activated fly ash-based geopolymer samples. This method was used by assimilation with standardized methods for characterizing the porosity of concrete, which allows the determination of this parameter based on the apparent and real density of the material. The porosity of the alkali-activated fly ash-based geopolymer samples was determined using the pycnometer method. The concrete samples were crushed and a representative amount was collected. The obtained material was placed in the mill and then passed through a 0.02 mm sieve. After sieving, it was dried at constant mass in the oven. The porosity of the samples was calculated by means of apparent density and real density, as a percentage.

The porosity of geopolymer materials was monitored and quantified to study the influence of this parameter on the mechanical properties, for the use of different types of aggregates.

For each physico-mechanical indicator experimentally determined for the alkali-activated fly ash-based geopolymer samples (density, flexural strength, compressive strength, porosity), a variation of the specified indicator was calculated and expressed as a percentage difference to the value obtained in the control sample (P1).

### 2.4. Optical, SEM and EDS Analysis of Samples

Aggregate distribution and porosity analysis were carried out on each sample by microscopic examination using a Leica SAPO optical stereomicroscope (LEICA, Wetzlar, Germany). From the overall sample set, three mixtures were selected for scanning electron microscopy (SEM) and energy-dispersive spectroscopy (EDS): P1—control sample, P5—granular class 0/4 mm recycled glass aggregates and P7—micronized quartz. The selection criteria included the mixtures with the most favourable physico-mechanical performances for recycled glass aggregates and the mixture with micronized quartz, which showed superior physico-mechanical performance compared to all quartz-type aggregate mixtures. Considering that a binder-aggregate ratio greater than 1:1 (1.25:1) typically results in an increased water absorption, it was important to balance the amount of available binder in the composite matrix to effectively incorporate the aggregates to achieve good mechanical strength performances; therefore, samples with the average binder to aggregate ratio of 1:1 were selected for the optical, SEM and EDS analyses.

The SEM and EDS images were acquired with a JEOL/JSM 5600-LV scanning electron microscope (JEOL Ltd., Tokyo, Japan) using the secondary electron imaging (SEI) mode at an acceleration voltage of 15 kV. As part of the preparation process, to increase the electrical conductivity for electron microscopy analysis, the samples were coated with gold by plasma sputtering.

The aim of using these methods was both to demonstrate the good conditions of the geopolymerization reaction process, with the formation of specific compounds, and to highlight their homogeneous distribution in the geopolymer matrix, with direct effects on the mechanical behaviour.

## 3. Results and Discussions

### 3.1. Physico-Mechanical Properties of the Samples

**Figure 5:**
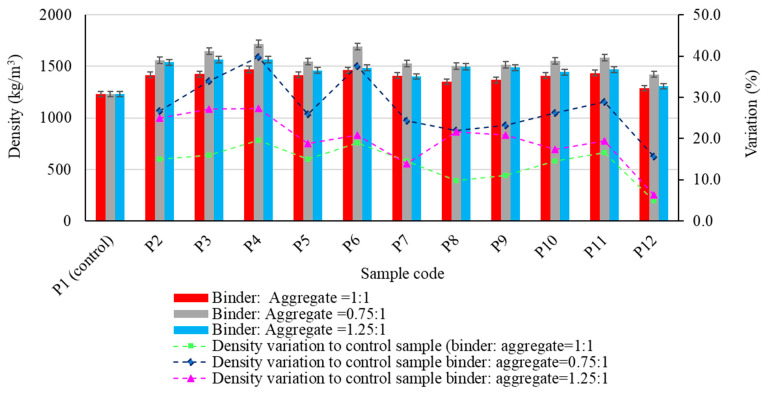
The influence of aggregates on the density of the alkali-activated fly ash-based geopolymer samples.

**Figure 6:**
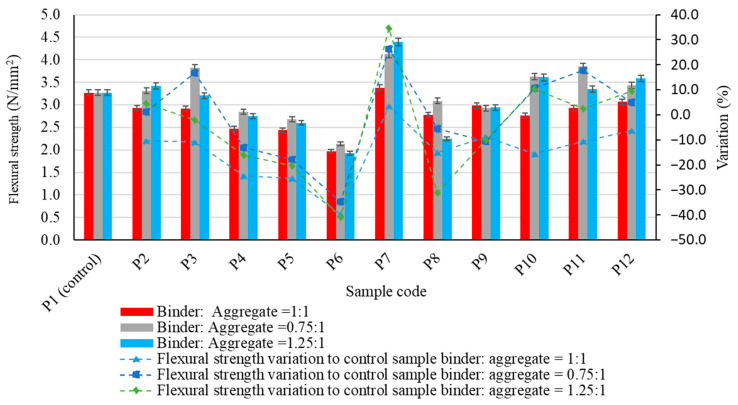
The influence of aggregates on the flexural strength of the alkali-activated fly ash-based geopolymer samples.

**Figure 7:**
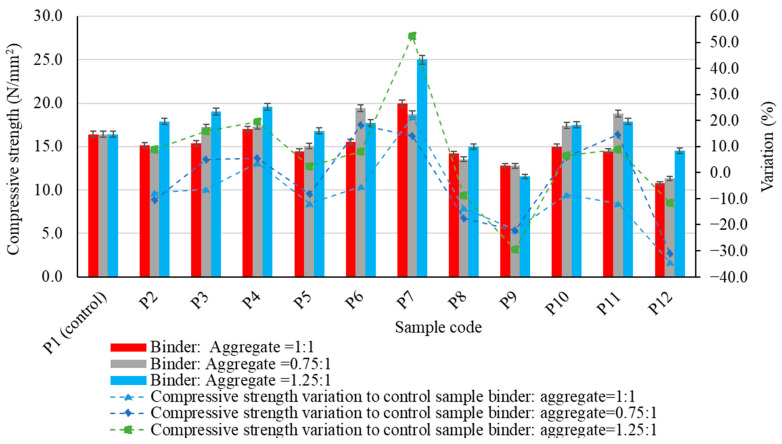
The influence of aggregates on the compressive strength of the alkali-activated fly ash-based geopolymer samples.

**Figure 8:**
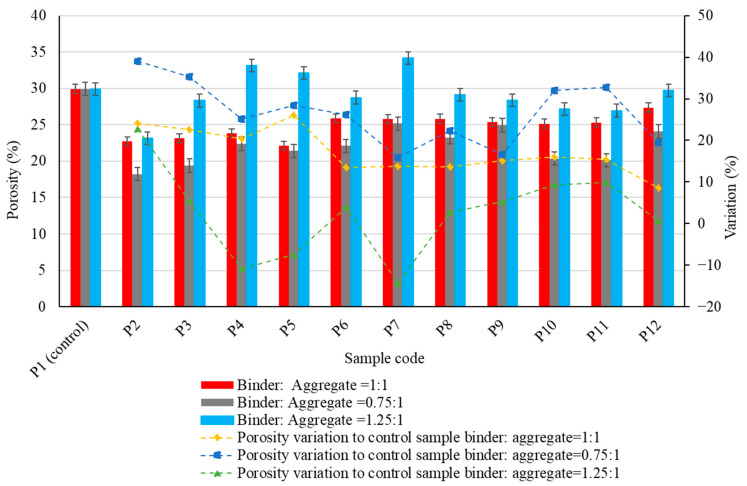
The influence of aggregates on the porosity of the alkali-activated fly ash-based geopolymer samples.

**Figure 9:**
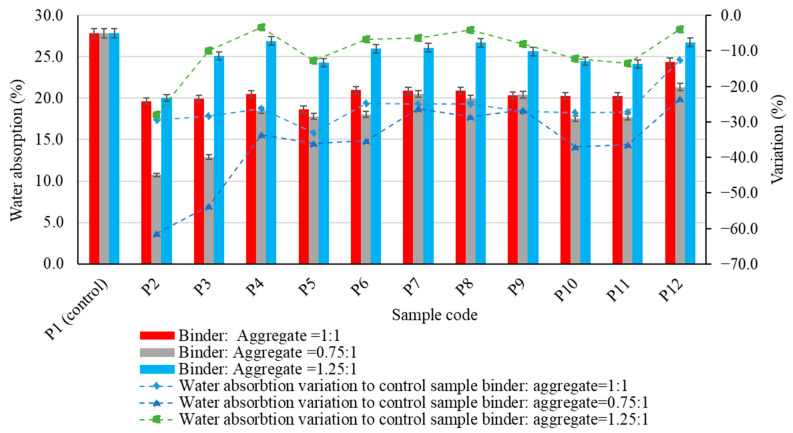
The influence of aggregates on water absorption of the alkali-activated fly ash-based geopolymer samples.

The experimental results regarding the physico-mechanical properties of the alkali-activated fly ash-based geopolymer samples are presented in Figure 5, Figure 6, Figure 7, Figure 8 and Figure 9, as the arithmetic mean of the individual values for each situation.

As observed in Figure 5, as expected, the density of the geopolymer samples increased when aggregates were incorporated in the geopolymer binder matrix, regardless of their type, compared to the control sample. However, results show that both the type of aggregate and the geopolymer binder to aggregate ratio influence the behaviour in the variation of this parameter. It can, therefore, be stated that irrespective of the geopolymer binder to aggregate ratio the maximum percentage increase in bulk density compared to the control sample was obtained when incorporating granular size 4/8 mm natural aggregates (Mixture P4). This increase was 19.55% for the geopolymer samples with a binder: aggregate ratio 1:1, 39.72% for 0.75:1 and 27.33% for 1.25:1. The lowest increase in bulk density, regardless of the geopolymer binder to aggregate ratio, was obtained for samples produced using spent garnet as the aggregate: 4.91% (binder: aggregate ratio 1:1), 15.65% (binder: aggregate ratio 0.75:1) and 6.41% (binder: aggregate ratio 1.25:1).

The impact on flexural strength of using different aggregates is shown in Figure 6. Results show that adding aggregates into the geopolymer binder matrix can have both a positive and a negative effect on this parameter, depending on the type of aggregate and the binder:aggregate ratio.

Thus, except for micronized quartz (P7), which showed a slight increase (3.47% with respect to the control sample), a reduction in flexural tensile strength was observed for all the types of aggregate used when their respective quantities were equal to that of the geopolymer in the composite matrix.

The maximum reduction in flexural tensile strength of geopolymer binder composites compared to the control sample was 37.72%. This was observed when using recycled glass aggregates of granular class 4/8 mm (P6), at a geopolymer binder to aggregate ratio of 1:1. As the amount of geopolymer binder in the composite matrix decreased (geopolymer binder to aggregate ratio of 0.75:1), the influence of the aggregate type and its granular class on the flexural tensile strength became more apparent. The parameter increases with respect to the control sample were recorded when polygranular sand (P2), natural aggregates granular class 0/4 mm (P3), micronized quartz (P7), granulated quartz 0/0.6 mm (P10), 0.3/0.7 mm (P11) or spent garnet (P12) were used. The maximum increase was 26.41% compared to the control sample; this was observed in the case of micronized quartz. Conversely, the use of coarse aggregates, natural aggregates of granular class 4–8 mm (P4) or recycled glass aggregates of granular class 4/8 mm (P6), and the effect of using recycled glass aggregates—including small-sized recycled glass aggregates of granular class 0/4 mm (P5)—leads to a decrease in flexural strength. The peak value is 34.80% (P6) compared to the control sample. As the amount of geopolymer binder in the composite matrix was increased (geopolymer binder to aggregate ratio of 1.25:1), different effects were also observed, both in terms of increase and decrease in flexural strength, depending on the type and granulation of the aggregate. In general, the positive effect, which results in an increase in the parameter studied, is maintained using granular sand or quartz. This is consistent with the previous scenario (geopolymer binder to aggregate ratio of 0.75:1). The most significant improvement was observed when micronized quartz (P7) was used. This resulted in an increase of 34.51% compared to the control sample. Similarly, the diminishing effect on flexural strength due to the incorporation of recycled glass aggregates into the geopolymer binder matrix is again evident, with the most significant reduction of 41.01% being observed for large-sized recycled glass aggregates of granular class 4/8 mm (P6).

When analysing the influence of the type of aggregate, its granulation and the geopolymer binder:aggregate ratio on the compressive strength of the geopolymer composites (Figure 7), several similarities can be observed when compared with the control sample (geopolymer binder without aggregates). Regardless of the geopolymer binder to aggregate ratio, the most significant reduction in compressive strength compared to the control is observed when using spent garnet (P12): 34.46%, 30.99% and 11.64%. In addition, the use of micronized quartz (P7) increased the compressive strength of the samples regardless of the geopolymer binder to aggregate ratio, with values of 21.47%, 14.21% and 52.38%. The use of quartz aggregates does not guarantee a consistent effect on compressive strength. Depending on the granulation of the aggregates, compressive strength may be improved or reduced. Even when using recycled glass aggregates, an increase in the compressive strength was observed. It should be noted that the granular class of the aggregates is a key element that can have either positive or negative effects. In some cases, the compressive strength of the composite can be increased by more than 18%, especially when these aggregates with a large grain size (4/8 mm) are introduced into the geopolymer binder matrix. However, this is at a reduced quantity (geopolymer binder to aggregate ratio = 0.75:1).

A cumulative analysis of the mechanical strength in both flexural and compressive strength tests for all geopolymer binder composites considering all types of aggregate used suggests that in most cases a geopolymer binder to aggregate ratio of 1:1 is not the most favourable alternative. Thus, in most situations, reducing the amount of binder by 25% (geopolymer binder to aggregate ratio = 0.75:1) resulted in an improvement in flexural strength, while maintaining the aggregate type.

However, there were instances where increasing the amount of binder by 25% (geopolymer binder to aggregate ratio = 1.25:1) resulted in an improvement in mechanical performances, particularly natural aggregates with a bigger particle size (4/8 mm). In the case of compressive strength, it was observed that an increase of 25% in the amount of binder (geopolymer binder to aggregate ratio = 1.25:1) can be a factor, with the potential to improve this performance, both for natural aggregates, recycled glass aggregates or quartz aggregates. It can, therefore, be seen that in terms of mechanical strength performance, similar to cementitious composites, both the binder matrix and the aggregate matrix structure of the composite, as well as their interaction and the bond between them, contribute to the overall result. Furthermore, as will be shown indirectly by the analysis of porosity and water absorption of the analysed samples, all these aspects are closely related to the porosity and distribution of pores within the composite mass.

The experimental results indicate the influence of introducing aggregates into the geopolymer binder matrix, but there is also an influence from the characteristics of these aggregates on the apparent porosity of the composites (Figure 8). As observed, in general, the porosity has a decreasing trend with the introduction of aggregates into the geopolymer binder matrix. This decreasing trend of porosity is generalized for the situations binder:aggregate = 1:1, respectively 0.75:1, regardless of the nature of the aggregates. In the case of a binder:aggregate ratio = 1.25:1, exceptions, i.e., increases in open porosity compared to the control sample, are observed for the situations P4 (natural aggregates granular class 4/8 mm), P5 (glass granular class 0/4 mm) and P7 (micronized quartz).

The increase in open porosity is probably due to various causes: in the case of natural aggregates, this is due to the larger grain size; in the case of glass aggregates, it is due to the shape of the grains and the lack of adhesion in the contact zone; and in the case of micronized quartz, it is probably due to the additional SiO_2_ input involved in the geopolymerization mechanism. For each type of aggregate, the same trend of porosity evolution is observed as a function of the binder:aggregate ratio, i.e., a higher amount of binder results in a higher open porosity, which is in correlation with the experimental results recorded for water absorption (a phenomenon that occurs through the capillarity of the material and is directly related to the open porosity).

Research clearly evidences that the water to solids ratio of the mixtures plays an important role in the pore size distribution of the cured geopolymers. In the case of geopolymerization, water is consumed only marginally during the alkali activation process of fly ash. For this reason, the volume fraction of the liquid activator governs the final open porosity of the geopolymers. Curing temperature and curing time also play an important role in the definition of geopolymer porosity. In general, a systematic increase in the total volume of pores is observed when the curing temperature increases. Inconsistent results have also been found in relation to the variation of porosity and pore size distribution during the curing time [90,91].

In terms of water absorption of the geopolymer binder composites (Figure 9), it is most evident that for all types of aggregates used, no specific behaviour could be observed for the results obtained when compared to the control sample. It can also be observed that a 25% reduction in the amount of geopolymer binder in the composite (geopolymer binder to aggregate ratio = 0.75:1) results in a reduction in water absorption, whereas an increase in the amount of geopolymer binder in the composite (geopolymer binder to aggregate ratio = 1.25:1) results in an increase in this parameter compared to the value obtained for the same binder to aggregate ratio.

The most significant changes were observed for the samples produced using polygranular sand (P2), in which a reduction in the quantity of geopolymer binder resulted in a reduction in water absorption of over 45%. Similarly, when using natural aggregates with a large grain size (P4), an increase in the amount of geopolymer binder resulted in a reduction in water absorption of more than 31% compared to the situation with a binder to aggregate ratio of 1:1. Analysis of the experimental data obtained for a constant binder to aggregate ratio shows the influence of the nature and granulometry of the aggregates on water absorption. Thus, for a binder to aggregate ratio of 1:1, the most significant reduction in water absorption compared to the control sample was recorded for the composite with recycled glass aggregates of granular size 0–4 mm (P5)—33.03%. However, this trend was not as pronounced when using granular class 4–8 mm recycled glass aggregates (P6), indicating that their distribution in the geopolymer binder matrix modifies the open porosity. This is a factor closely related to water absorption. It is probable that recycled glass aggregates with a small grain size allow a better distribution in the mass, together with a lower open porosity; or even the possibility of the fine part of the aggregate closing some of the open pores formed in the geopolymer binder during the geopolymerization process. This hypothesis is supported by the behaviour of composites produced using polygranular sand (P2), natural aggregates of granular class 0–4 mm (P3) or quartz aggregates; these are all characterized by a higher proportion of fine particles and a significant reduction in water absorption. Furthermore, when examining the results in which the amount of geopolymer binder in the composite was reduced (geopolymer binder to aggregate ratio = 0.75:1), there was an enhanced effect in reducing water absorption. This exceeded a 60% reduction compared to the control sample when using polygranular sand (P2). Conversely, when the amount of geopolymer binder in the composite was increased (geopolymer binder to aggregate ratio = 1.25:1), the effect on reducing water absorption was less pronounced. These results underline both the importance and the feasibility of reducing water absorption in composites through key factors: the type of aggregate, granularity and the amount of binder available in the matrix.

### 3.2. Optical, SEM and EDS Analysis of Samples

In order to assess the behaviour of the alkali-activated fly ash-based geopolymer samples, the variation of the observed physico-mechanical indicators was further supported by means of optical analysis and microstructural characterization of the samples.

**Figure 10:**
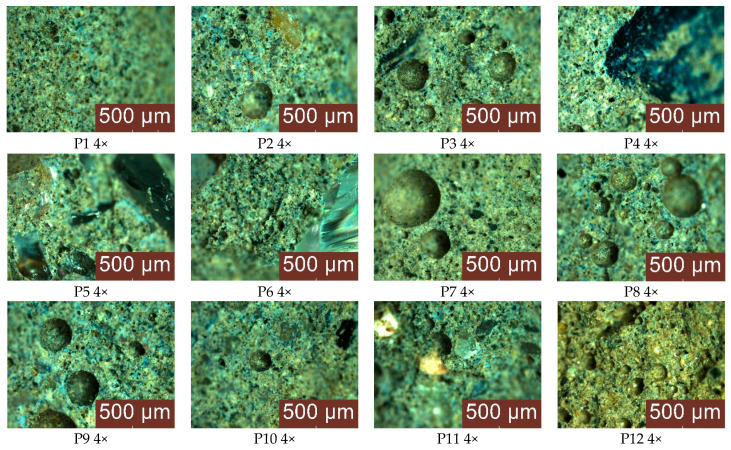
Optical microscopy images of the control sample and geopolymer binder composites for binder:aggregate ratio = 1:1 (4× measurement).

Microscopic analysis of the structure of the geopolymers (Figure 10) has highlighted mixtures in which the distribution of the aggregates is homogeneous and uniform. An exception is seen when using natural or recycled glass aggregates with large particle sizes (P4) and (P6), which show a tendency towards segregation. In addition, the presence of pores formed during the setting process was observed in the mass, with a variable distribution and size depending on the type and granular class of the aggregates. The control sample has a porosity characterized by an even distribution of numerous small pores. In contrast, the composite samples show a non-uniform distribution of porosity, with the presence of larger pores (maximum diameter, 1140 μm, for sample P7) but in smaller quantities. The use of recycled glass aggregates tends to reduce porosity, both in terms of pore size and frequency. In this case, the behaviour of the geopolymer samples is attributed to a combination of effects: improvement due to reduced porosity, but also lack of improvement due to the tendency for segregation, with the aggregates migrating towards the lower part of the specimens; this is probably due to a reduced degree of cohesion with the geopolymer binder. Quartz aggregates generally tend to reduce porosity but depending on their granulometry the composite matrix may have areas of clustered pores along with more compact areas of few and small pores. Spent garnet has a comparable effect on porosity, such as the patterns observed with coarse sand and natural aggregates, where the composite matrix presents a combination of larger and smaller pores which are randomly distributed.

**Figure 11:**
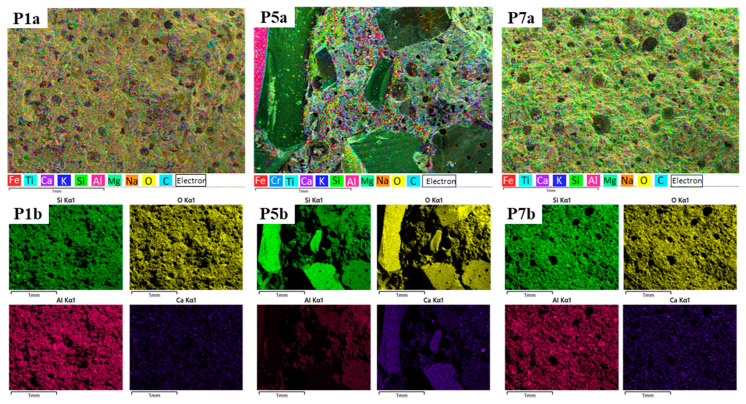
Elemental distribution map of Si, O, Ca and Al in the control sample (P1(a,b)) and the samples with recycled glass aggregates granular class 0/4 mm (P5(a,b)) and micronized quartz (P7(a,b)) for binder:aggregate ratio = 1:1.

The morphology and microstructure of the alkali-activated fly ash geopolymer composites were investigated by analysing sections of samples P1, P5 and P7 using SEM and EDS techniques. Figure 11 P1a, P5a and P7a show the SEM images at ×50 magnification, while Figure 11 P1b, P5b and P7b show the elemental distribution maps in the samples investigated. Figure 11 P1a shows the SEM image of the sample corresponding to the control composition. Visual assessment of the region examined suggests the formation of a dense structure with uniform micro-pore distribution within the sample. The average micro-pore size measured within the region analysed was 61.2 μm. A comparable structure was observed in sample P7. However, in this instance, the average micro-pore size measured approximately 80.8 μm. However, in Figure 11 P5a, which illustrates the sample containing recycled glass aggregates (granular class 0/4 mm), a distinct scenario emerges. The glass aggregates appear embedded in the geopolymer binder mass, which appears less cohesive and more porous.

**Figure 12:**
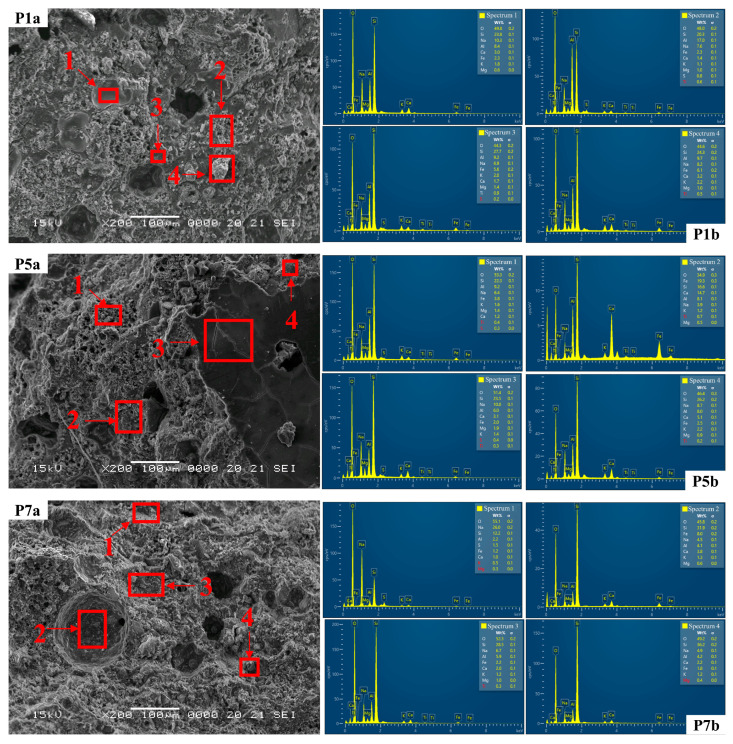
SEM micrographs and EDS spectra, recorded in the selected marked area (1–4), of the P1a (control composition), P5a (recycled glass) and P7a (micronized quartz) samples; for binder:aggregate ratio = 1:1.

The comprehensive EDS spectra (Figure 12 P1b, P5b and P7b and Table 3) as well as the EDS elemental distribution maps (Figure 11 P1b, P5b and P7b) reveal a uniform distribution of Si, Na and Al over the entire area examined in each specimen. This is consistent across all specimens. These elements play a direct role in the geopolymerization reactions that form robust Si-Al and Na-Al-Si bonds and, therefore, provide a basis for the strength of the geopolymer composite. The elevated presence of O, Si, Al and Na in distinct regions of the samples, correlated with the observed microstructures, suggests the presence of sodium alumino-silicate hydrate (N-A-S-H) gel. Conversely, in the case of P5, concentrations of Si and O are heightened in regions where glass aggregates are present (attributed to the specific composition of glass: CaO, Al_2_O_3_ and SiO_2_), while Al distribution is observable.

**Figure 13:**
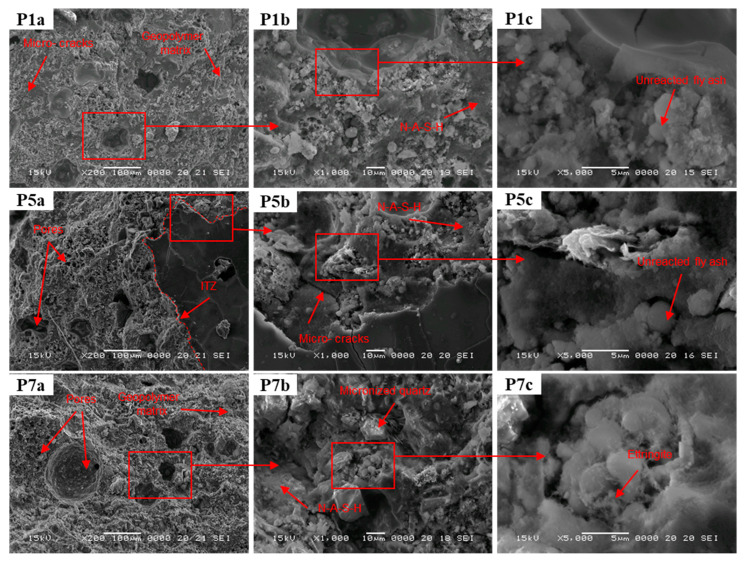
SEM images of the control sample (P1) and the samples containing glass aggregate (P5) and micronized quartz (P7), respectively, at magnifications of ×200 (P1a, P5a and P7a), ×1000 (P1b, P5b and P7b) and at ×5000 (P1c, P5c and P7c); for binder:aggregate ratio = 1:1. The areas recoded at higher magnification are marked in the low magnification image. ITZ—Interfacial Transition Zone.

The SEM images were also used to determine the influence of glass and micronized quartz aggregates on the morphology and surface topography of the geopolymer composites obtained from alkali-activated fly ash, as shown in Figure 13 control sample, at magnifications of ×200 (Figure 13 P1a), ×1000 (Figure 13 P1b) and ×5000 (Figure 13 P1c), the sample containing glass aggregates (0/4 mm) at magnifications of ×200 (Figure 13 P5a), ×1000 (Figure 13 P5b) and ×5000 (Figure 13 P5c), and the sample containing micronized quartz at magnifications of ×200 (Figure 13 P7a), ×1000 (Figure 13 P7b) and ×5000 (Figure 13 P7c).

The main features observed in the SEM images of the three samples analysed include a compact distribution of fully reacted, partially reacted or unreacted fly ash particles, a coherent and homogeneous mass encapsulating and binding the particles (the geopolymer binder), and networks of intergranular and intragranular pores [81]. Thus, the SEM images reveal that sample P7 exhibits a more granular appearance, which is characterized by larger-diameter pores that are fewer in number compared to specimens P1 and P5. Although a well-developed geopolymer matrix is observed for sample P5, it appears to be less homogeneous and uniform than that observed for P1 and P7, which can be attributed to the inclusion of the glass aggregate. Furthermore, the glass aggregate appears to be well embedded and encapsulated within the geopolymer binder, with little to no porosity or voids surrounding the aggregate particles. In addition, all samples show small cracks at low magnification.

Upon closer examination of all three samples (Figure 13 P1c, P5c and P7c), the images indicate the presence of particles with smooth surfaces that correspond to the morphology of barely reactive class F fly ash, which appears to be surrounded by a layer of a fine needle-shaped material presumably composed of unreacted alkali that precipitated during the process and formed these stripe-like structures. The general morphology of the geopolymer composites shows densely packed particles with most of the small cenospheres likely dissolved and the amorphous phase, identified as alkaline aluminium silicate hydrate gel (N-A-S-H), surrounding the embedded aggregates. Figure 13 P7b details the aspect of the sample containing micronized quartz at a magnification of ×1000. Two types of angular and irregular shaped particles can be observed. The bulky, isolated crystals correspond to the micronized quartz that is utilized as an aggregate in this specimen, while the hexagonal prism crystals and partially acicular particles can be attributed to the incomplete dissolution of the fly ash.

## 4. Conclusions

The choice of aggregates in geopolymer formulations depends on various factors, including local availability, the desired properties of the final product, and the specific application of the geopolymer. Experimentation and optimization of mix designs are often necessary to achieve the desired balance of strength, workability and durability in geopolymer concrete. The use of recycled aggregates or industrial by-products as alternative aggregate aligns with sustainability goals by reducing the environmental impact of construction materials.

This study investigated the effect of different aggregates on alkali-activated fly ash geopolymer composites, produced using Romanian local materials, and shows that density increases with the addition of aggregates, especially with 4–8 mm natural aggregates; this is influenced by both aggregate type and geopolymer binder to aggregate ratio. The introduction of aggregates has variable effects on flexural tensile and compressive strength, with results dependent on the type and particle size distribution of the aggregate and the binder to aggregate ratio. Flexural tensile strength varies, decreasing with recycled glass aggregates and increasing with micronized quartz. A 25% reduction in binder generally improves flexural strength, while a 25% increase in binder can improve compressive strength. This demonstrates the interplay between binder matrix, aggregate structure and their interaction. In addition, water absorption in geopolymer composites decreases significantly as the amount of binder is reduced, highlighting the key role of these factors in shaping composite properties.

These results are supported by the structural and microstructural characterization of the samples by optical, SEM and EDS images. These images show a largely homogeneous distribution of Si, Na and Al in the geopolymer composites, and a relatively uniform distribution of macro- and micro-pores; therefore, this contributes to the physico-mechanical properties of alkali-activated fly ash geopolymer composites.

The particle size distribution of aggregates can affect the workability, strength and density of geopolymer-based materials. A well-graded aggregate mix is generally preferred to optimize packing density and improve mechanical properties. The type of aggregate can also influence the porosity and permeability of the geopolymer composite. Porous aggregates may increase water absorption, which affects the durability of the material. Therefore, selecting aggregates with low porosity is desirable for improved performance.

Aggregates contribute significantly to the mechanical properties of geopolymer composites. The type and quality of the aggregate can impact the compressive and flexural strength, as well as the hardness of the final material.

Considering environmental aspects, recycled aggregates or industrial by-products may be used as alternatives to traditional natural aggregates, aligning with sustainable construction practices.

Therefore, optimizing the combination of aggregates and other components is essential to achieve the desired properties in geopolymer materials. Trial-and-error programmes and testing are often required to fine-tune mix designs for specific applications and performance requirements. Further studies will be conducted with specific mix-design optimization with variations in molar concentration of the NaOH solution, Na_2_SiO_3_/NaOH solutions ratio and binder to aggregate ratio.

## Figures and Tables

**Table 1:** Characterization of fly ash.

Oxide composition (%)	SiO_2_	Al_2_O_3_	Fe_2_O_3_	CaO	MgO	SO_3_	Na_2_O	K_2_O	P_2_O₅
	46.94	23.83	10.08	10.72	2.63	0.45	0.62	1.65	0.25
	TiO_2_	Cr_2_O_3_	Mn_2_O_3_	ZnO	SrO	CO_2_	P.C.	SiO_2_+Al_2_O_3_	
	0.92	0.02	0.06	0.02	0.03	-	2.11	70.77	
R_0.045_ (%)	31.40								

**Table 2:** Bulk apparent density of the aggregates used in production of the alkali-activated fly ash-based geopolymer samples.

Aggregate Type	Polygranular Sand	Natural Aggregates 0/4 mm	Natural Aggregates 4/8 mm	Glass Aggregate Reciclată 0/4 mm	Glass Aggregate Reciclată 4/8 mm	Micronized Quartz	Granulated Quartz 0/0.3 mm	Granulated Quartz 0/0.5 mm	Granulated Quartz 0/0.6 mm	Granulated Quartz 0.3/0.7 mm	Spent Garnet
Bulk apparent density (kg/m^3^)	1660	1580	1530	1350	1430	1030	1430	1440	1450	1420	2690

**Table 3:** Spectra values of EDS for various distributions of elements in the examined regions (1–4).

Mixture	Spectra	O %	S %i	Na %	Al %	Ca %	Fe %	K %	Mg %
**P1a**	S1	49.8	23.8	10.3	8.4	3	2.3	1.8	0.6
	S2	48	20.3	7.6	17	1.4	2.3	1.1	1
	S3	44.3	27.7	6.9	9.2	1.7	5.6	2	1.4
	S4	44.6	24.3	8.2	9.7	3.2	6.1	2.2	1
**P5a**	S1	53.3	22.3	6.4	9.2	1.2	3.8	1.6	1.4
	S2	34.9	16.6	3.9	8.1	14.7	19.3	1.2	0.5
	S3	51.4	23.5	10	6	3.1	2	1.4	1.9
	S4	46.4	26.2	8.7	8	5.1	2.5	2.2	0.9
**P7a**	S1	55.1	12.2	26	2.2	1	1.2	0.5	0.3
	S2	45.8	31.9	4.5	4.1	3.8	8	1.3	0.6
	S3	52.3	28.5	6.7	5.9	2	2.2	1.2	1
	S4	49.2	36.2	4.9	4.2	2.2	1.8	1.2	0.4

## Data Availability

Data are contained within the article.
